# Association between birthweight discordance and extrauterine growth restriction among preterm twins: a national multi-center study in China

**DOI:** 10.3389/fped.2025.1709824

**Published:** 2026-01-23

**Authors:** Qian Chen, Bijun Shi, Lizi Lin, Danfang Lu, Jiayi Zhang, Shuhua Ren, Kang Huang, Wei Shen, Zhifeng Chen, Jin Liu, Chunming You, Guifang Li, Hong Jiang, Hongping Rao, Jianwu Qiu, Xian Wei, Yayu Zhang, Xiaobo Lin, Haiyan Jiang, Shasha Han, Fan Wang, Weixing Zhang, Xiufang Yang, Yitong Wang, Niyang Lin, Xiaohua Tan, Qiliang Cui

**Affiliations:** 1 Department of Child Healthcare, Guangdong-Hong Kong-Macao Greater Bay Area Higher Education Joint Laboratory of Maternal-Fetal Medicine, Guangdong Provincial Key Laboratory of Major Obstetric Diseases, Guangdong Provincial Clinical Research Center for Obstetrics and Gynecology, The Third Affiliated Hospital, Guangzhou Medical University, Guangzhou, Guangdong, China; 2 Joint International Research Laboratory of Environment and Health, Ministry of Education, Guangdong Provincial Engineering Technology Research Center of Environmental Pollution and Health Risk Assessment, Department of Occupational and Environmental Health, School of Public Health, Sun Yat-sen University, Guangzhou, Guangdong, China; 3 Department of Pediatrics, Peking University Third Hospital, Beijing, China; 4 The First Clinical Medical College of Kunming Medical University, Kunming Medical University, Kunming, Yunnan, China; 5 Department of Neonatology, Sichuan Jinxin Xinan Women & Children’s Hospital, Chengdu, Sichuan, China; 6 Department of Neonatology, Affiliated Hospital of Guizhou Medical University, Guiyang, Guizhou, China; 7 Department of Neonatology, Women and Children’s Hospital, School of Medicine, Xiamen University, Xiamen, Fujian, China; 8 Department of Neonatology, The Tenth Affiliated Hospital of Southern Medical University, Dongguan, Guangdong, China; 9 Department of Neonatology, The First Affiliated Hospital of Shaoyang University, Shaoyang, Hunan, China; 10 Department of Neonatology, Guangdong Second Provincial General Hospital, Guangzhou, Guangdong, China; 11 Department of Neonatology, Cangzhou People’s Hospital, Cangzhou, Hebei, China; 12 Department of Neonatology, Affiliated Hospital of Yan an University, Yan’ an, Shanxi, China; 13 Department of Neonatology, Huizhou Central People’s Hospital, Huizhou, Guangdong, China; 14 Department of Neonatology, The Affiliated Yue Bei People’s Hospital of Shantou University Medical College, Shaoguan, Guangdong, China; 15 Department of Neonatology, Xiaogan Hospital Affiliated to Wuhan University of Science and Technology, Xiaogan, Hubei, China; 16 Department of Neonatology, The Affiliated Hospital of Inner Mongolia Medical University, Hohot, Inner Mongolia, China; 17 Department of Neonatology, The Second Affiliated Hospital of Shantou University Medical College, Shantou, Guangdong, China; 18 Department of Neonatology, Affiliated Hospital of Guizhou Medical University, Baotou, Inner Mongolia, China; 19 Department of Neonatology and Pediatrics, The First Affiliated Hospital of Jinan University, Guangzhou, Guangdong, China; 20 Department of Neonatology, Lanzhou University Second Hospital, Lanzhou, Gansu, China; 21 Department of Neonatology, Xinxiang Central Hospital, Xinxiang, Henan, China; 22 Department of Neonatology, Neonatology Department, Zhongshan City People’s Hospital, Zhongshan, Guangdong, China; 23 Department of Neonatology, The Binhaiwan Central Hospital of Dongguan, Dongguan, Guangzhou, China; 24 Department of Neonatology, The First Affiliated Hospital of Shantou University Medical College, Shantou, Guangdong, China; 25 Department of Neonatology, The Third Affiliated Hospital, Guangzhou Medical University, Guangzhou, Guangdong, China

**Keywords:** birthweight discordance, extrauterine growth restriction, small gestation age, twin, NICU

## Abstract

**Background:**

This multicenter study investigated the association between birthweight discordance (BWD) and extrauterine growth restriction (EUGR) in preterm twins, and evaluated the modifying role of small for gestational age (SGA).

**Methods:**

Data from 2,496 infants (1,248 twin pairs) admitted to 22 Chinese NICUs (2018–2020) were analyzed. BWD was calculated as the percentage difference in birthweight between larger and smaller twins, categorized into four groups (≤15%, 15%–20%, 20%–25%, >25%). EUGR was defined as discharge weight below the 10th percentile for corrected gestational age and sex (Fenton's chart). A generalized linear mixed model was employed to analyze the association between BWD and EUGR. Modification analysis was performed to assess the effect of SGA on this association.

**Results:**

BWD of >25% was associated with a significantly increased risk of EUGR compared to BWD ≤15% (adjusted OR = 1.59, 95% CI: 1.05–2.41). Stratified analysis revealed a consistent association in SGA infants (OR = 1.38, 95% CI: 1.30–1.47).

**Conclusion:**

Findings highlight BWD as a critical risk factor for EUGR, particularly in SGA twins. This association suggests that future research should investigate whether tailored monitoring and nutritional interventions in NICUs could help mitigate these growth disparities.

## Introduction

Twin pregnancies account for approximately 2%–4% of all births and have significantly increased globally, particularly in middle-income and developing countries (1, 2). In China, the twinning rate rose from 2.84% to 3.22% between 2012 and 2020 (3). This trend carries substantial public health implications, as over half of all twin births are preterm (4), and twin pregnancies are associated with a threefold to sevenfold higher risk of perinatal morbidity and mortality compared to singleton pregnancies (5).

Birthweight discordance (BWD) is a key determinant of postnatal outcomes in twin pregnancies (6), typically defined as a birth weight difference of ≥20%–25% between the larger and smaller twin (7). It is a well-established risk factor for pregnancy outcomes, including preterm birth, and increased neonatal intensive care unit (NICU) admissions, and perinatal morbidity such as bronchopulmonary dysplasia (BPD), neonatal necrotizing enterocolitis (NEC), persistent patent ductus arteriosus (PDA) (8), and neonatal sepsis. Recent cohort studies have also linked BWD to adverse long-term developmental consequences (9–11). Improving the long-term prognosis for twins with significant BWD has become a new focus of research. Current evidence suggests that even short-term relative malnutrition during sensitive periods can significantly adversely affect subsequent development (12, 13). Extrauterine growth restriction (EUGR) describes inadequate growth during the sensitive postnatal period up to 40 weeks' corrected gestational age. This condition is consistently linked to adverse neurodevelopmental outcomes at 24 months (14–16).

EUGR is defined as the growth of preterm infants falls below the expected weight based on intrauterine growth rates [i.e., ≤10% of the expected weight for their gestational age (17)], which is still a serious problem among NICUs preterm infants (18). The pathophysiology of EUGR is multifactorial, with key risk factors including low birthweight, delayed achievement of full enteral feeding (FEF), BPD, and NEC (17, 19–21). These very conditions are frequently associated with BWD (8), suggesting a potential link between BWD and EUGR. Previous studies have described the growth patterns among discordant twins. Xiang et al. found the smaller infants among discordant twins had a higher risk of growth restriction in the first 6 months after birth (22), which was consistent with other research (23). However, the evidence of previous studies was insufficient to confirm the association between BWD and EUGR, due to the small sample sizes, different cut-off levels of BWD, and great heterogeneity. Therefore, the correlation between BWD and EUGR remains an unanswered question.

The relationship is further complicated by the high prevalence (25%–35%) of small for gestational age (SGA) infants in twins (24). SGA is a risk factor for EUGR in singletons, suggesting a persistence of poor growth from the intrauterine to the extrauterine environment (25, 26). While epidemiological studies reported inconsistent relationships of BWD with SGA among twins. Our previous study found higher rates of SGA in twins with higher BWD among 4,011 twin pairs (27). Another multicenter cohort study showed no association between BWD with SGA in 939 twins (28). The role of SGA in the association between BWD and EUGR remains unclear.

Using data from a nationwide multicenter study with NICU data, we propose two hypotheses: (1) higher BWD increases the odds of EUGR, and (2) SGA modifies the association between BWD and EUGR.

## Methods

### Study population and overall design

This national cross-sectional study, established by the Chinese Multicenter Birthweight Discordance Twins Collaborative Group (CBDC group), included 22 hospitals in 15 cities across 12 provinces/municipalities in China. All hospitals were government-designated tertiary class-A, with 20 affiliated with universities. Initially, a standardized protocol was developed for a pilot multi-center study in six cities in Guangdong Province. This protocol was later applied to other study sites. Detailed geographical distribution and specific demographic characteristics of participating centers are shown in Supplementary Figure S1. Trained data abstractors at each site collected patient data from medical records, ensuring patient identity remained confidential. Data were entered directly into a customized database (EpiData 3.1) with built-in error checking and a standard operation manual. The data were electronically transmitted to the coordinating center at the Third Affiliated Hospital of Guangzhou Medical University for integration and analysis. The CBDC database includes all live twin births from January 1, 2018, to December 31, 2020. This study conducted a secondary analysis of CBDC data for preterm twins admitted to participating NICUs with hospital stays of ≥7 days. From the initial 1,758 pairs of preterm twins in NICUs, we excluded 137 pairs with congenital malformations or genetic metabolic diseases, 58 pairs with twin to twin transfusion syndrome or twin anemia-polycythemia sequence, 66 pairs of monochorionic monoamniotic twins, and 249 pairs without discharge weight data (see Supplementary Figure S2). Finally, 1248 twin pairs (2,496 individuals) were eligible for analysis.

### Criteria for discharge

Infants were considered eligible for discharge upon meeting all of the following criteria, which from the guidelines of the American Academy of Pediatrics (29): 1) Physiological stability without respiratory support, and freedom from clinically significant apnea and bradycardia for at least 5 days; 2) Ability to maintain normal body temperature in an open crib; 3) Consistent weight gain on full oral feeding without the need for supplemental tube feeding; 4) Resolution of major acute diseases.

### Definitions

BWD was defined as the percentage difference in birthweight between the larger and smaller twin using the formula: (larger birthweight - smaller birthweight)/larger birthweight × 100%, with a cut-off of >20%. Participants were categorized into four groups: ≤15%, >15%–20%, >20%–25%, and >25%, as previous studies (30). EUGR was defined as a discharge weight below the 10th percentile for the same corrected gestational age and gender, according to the Fenton growth chart (31). SGA was defined as birthweight below the 10th percentile for Chinese twins, specific to gestational age (GA) and chorion (32). Diagnoses of BPD, NEC, PDA, and neonatal sepsis were established by Practical Neonatology (5th edition) (33). The standardized feeding protocol was developed following the *Clinical Application Guidelines for Neonatal Nutritional Support in China* (34). FEF was defined as enteral feeding volume of 150 ml per kilogram of body weight per day, or 110 kcal per kilogram of body weight per day, or exclusive breast-feeding, whichever occurred first (35). Weights at birth and discharge were converted to z-score using the Fenton growth charts. To better evaluate the longitudinal growth of the infants, the difference between discharge and birth weight *z*-scores (Δ*z*-score) was calculated.

### Covariates

The collected data included maternal age (<35 years, ≥35 years), education level (high school and below, college degree and above), multiparity, use of assisted reproductive technology (ART), cesarean delivery, twin chorionicity (dichorionic diamniotic (DCDA), monochorionic diamniotic (MCDA)), and complications such as gestational diabetes (GDM) and hypertensive disorder complicating pregnancy (HDP). Neonatal data included GA (<31^+6^ weeks, 32–36^+6^ weeks) and complications like BPD, NEC, PDA, or neonatal sepsis. Nutritional information included the time to achieve of FEF and the duration of hospital stay.

### Statistical analysis

We calculated mean (SD) or median (IQR) values for continuous variables and percentages for categorical variables. Characteristics were compared across BWD groups using rank sum or χ^2^ tests as appropriate.

Cases with any missing value in the demographic characteristics, adjusted confounders were excluded from the multivariate analysis. Of the 2,496 individuals, 15(0.60%) had incomplete data and were removed. We explored the association between BWD (both continuous and categorical) and EUGR by treating twins as separate individuals. To account for within-twin correlations, a generalized linear mixed model was used, treating twin pairs and hospitalization area as random effects (36). The main model included maternal age, GDM, HDP, ART, chorionicity, GA, infant gender, complications, FEF days, and hospital stay duration as fixed effects. Multicollinearity was assessed by calculating the Variance Inflation Factor (VIF) for each predictor. A VIF >10 was considered indicative of severe multicollinearity that required remediation (37), All VIF values in our model were below 3. The potential effect modification of SGA on the BWD-EUGR association was evaluated with multiplicative interaction terms in the model, adjusted for the same covariates. Stratified analysis based on the SGA infants was performed.

Sensitivity analyses included: (1) using a generalized additive model (GAM) to assess non-linear associations (38); (2) using stratified analyses by maternal and neonatal characteristics (maternal age, chorioamniotic sac type, GDM, HDP, GA and neonatal complications) with interaction terms; and (3) using ordinal regression with random effects to analyze the BWD-EUGR association, considering geographical birth areas as random effects, and twins without EUGR were the reference category.

Statistical analyses were performed using R version 4.2.2. All tests were 2-sided, with *P* < 0.05 considered significant.

## Results

### Characteristics of study subjects

The study population comprised 2,496 individuals with twin pregnancies: 1,772 (70.99%) infants with BWD ≤15%, 246 (9.86%) infants with BWD >15%–20%, 196 (7.85%) infants with BWD >20%–25% and 282 (11.30%) infants with BWD >25%. And 1,137 (45.55%) of them were diagnosed with EUGR. Infants with higher BWD had higher rates of cesarean delivery, MCDA, HDP, SGA, EUGR and neonatal complications than those with lower BWD. No statistical differences were observed among advanced maternal age, mother education levels, GA, infant's gender and other characteristics across different degree of BWD (*P* > 0.05) (Table 1).

**Table 1:** Characteristics of study participants (infants).

General characteristics	Overall, No. (%) (*N* = 2,496)	BWD (%), No.(%)				*P*-value
		≤15 (*n* = 1,772)	>15–20 (*n* = 246)	>20–25 (*n* = 196)	>25 (*n* = 282)	
Maternal characteristics						
Advanced maternal age	536 (21.49)	366 (20.68)	56 (22.76)	46 (23.47)	68 (24.11)	0.47
Mother education						
High school and below	1,600 (64.10)	1,112 (62.75)	164 (66.67)	140 (71.43)	184 (65.25)	0.08
College degree and above	896 (35.90)	660 (37.25)	82 (33.33)	56 (28.57)	98 (34.75)	
Multipara	936 (37.53)	682 (38.53)	102 (41.46)	50 (25.51)	102 (36.17)	<.01
ART	1,408 (56.68)	1,044 (59.18)	134 (54.92)	110 (56.70)	120 (42.55)	<.001
Cesarean delivery	2,142 (85.96)	1,498 (84.73)	208 (84.55)	182 (92.86)	254 (90.07)	<.01
Chorioamniotic sac type						
MCDA	742 (29.73)	492 (27.77)	82 (33.33)	62 (31.63)	106 (37.59)	<0.01
DCDA	1,754 (70.27)	1,280 (72.23)	164 (66.67)	134 (68.37)	176 (62.41)	
Pregnancy complications						
GDM	532 (21.38)	410 (23.22)	44 (17.89)	32 (16.33)	46 (16.43)	0.01
HDP	488 (19.61)	288 (16.31)	74 (30.08)	42 (21.43)	84 (30.00)	<.001
Neonatal characteristics						
GA, week (mean ± SD)	33.70 ± 2.19	33.68 ± 2.26	33.71 ± 2.19	33.90 ± 1.98	33.71 ± 1.93	0.60
GA composition						
<31^+6^w	450 (18.03)	324 (18.28)	50 (20.33)	30 (15.31)	46 (16.31)	0.48
32–36^+6^w	2,046 (81.97)	1,448 (81.72)	196 (79.67)	166 (84.69)	236 (83.69)	
Gender						
Boy	1,360 (54.49)	940 (53.05)	147 (59.76)	112 (57.14)	161 (57.09)	0.14
Girl	1,136 (45.51)	832 (46.95)	99 (40.24)	84 (42.86)	121 (42.91)	
SGA	354 (14.18)	146 (8.24)	39 (15.85)	48 (24.49)	121 (42.91)	<.001
Δ*z*-score	−0.77 (0.54)	−0.78 (0.47)	−0.73 (0.82)	−0.74 (0.44)	−0.76 (0.68)	0.51
EUGR	1,137 (45.55)	742 (41.87)	121 (49.19)	104 (53.06)	170 (60.28)	<.001
Neonatal complications	398 (22.46)	69 (28.05)	37 (18.88)	92 (32.62)	596 (23.88)	<.001
BPD	138 (7.79)	17 (6.91)	9 (4.59)	27 (9.57)	191 (7.65)	0.23
NEC	71 (4.01)	14 (5.69)	4 (2.04)	15 (5.32)	104 (4.17)	0.20
PDA	208 (11.74)	44 (17.89)	23 (11.73)	47 (16.67)	322 (12.90)	0.01
Neonatal sepsis	131 (7.39)	18 (7.32)	6 (3.06)	25 (8.87)	180 (7.21)	0.10
Nutritional information						
Start days of FEF, median (IQR), days	10.00 (7.00)	9.00 (9.00)	10.00 (10.00)	13.00 (11.00)	10.00 (9.00)	<.001
Duration of hospital stays, median (IQR), days	13.00 (14.00)	14.00 (16.00)	16.00 (16.00)	19.50 (20.75)	14.00 (16.00)	<.001

BWD, birth weight discordance; ART, assisted reproductive technology; MCDA, monochorionic diamniotic; DCDA, dichorionic diamniotic; GDM, gestational diabetes mellitus; HDP, hypertensive disorders of pregnancy; GA, gestational age; SGA, small for gestational age; EUGR, extrauterine growth restriction; BPD, bronchopulmonary dysplasia; NEC, neonatal necrotizing enterocolitis; PDA, patent ductus arteriosus; FEF, full enteral feeding.

### Association between BWD and EUGR

Table 2 presents the ORs for the association between BWD, both as a continuous variable and in categories, and the risk of extrauterine growth restriction (EUGR). The twin pairs-level random intercept variance was 1.29 [intraclass correlation (ICC) = 0.26] and the hospitalization area-level random intercept variance was 0.32 (ICC = 0.07). After adjusting for potential confounding factors, each 1% increment in BWD was associated with a 1.02-fold increase (95% CI: 1.00, 1.03) in the risk of individual EUGR. Additionally, BWD was subdivided into four distinct categories to further investigate this relationship. Compared to a BWD of ≤15%, the adjusted OR of EUGR for a BWD >25% was 1.59 (95% CI: 1.05, 2.41), with a significant p value for trend (*P*_for trend_ < 0.01).

**Table 2:** Associations between BWD and individual EUGR.

BWD	Crude model^a^			Adjusted model^b^		
	OR (95% CI)	*P*-value	*P*_for trend_	OR (95% CI)	*P*-value	*P*_for trend_
An increase in BWD of 1%	1.03 (1.02, 1.04)	<0.01	**–**	1.02 (1.00, 1.03)	0.01	**–**
Categories of BWD						
≤15%	Ref	**–**	<0.01	Ref	**–**	0.01
15%–20%	1.48 (1.01, 2.16)	0.04		1.50 (0.97, 2.31)	0.07	
20%–25%	1.82 (1.19, 2.77)	0.01		1.62 (1.01, 2.61)	0.04	
>25%	2.64 (1.84, 3.79)	<0.01		1.59 (1.05, 2.41)	0.03	

BWD, birth weight discordance; EUGR, extrauterine growth restriction; OR, odds ratio; ART, assisted reproductive technology; GDM, gestational diabetes mellitus; HDP, hypertensive disorders of pregnancy; GA, gestational age; FEF, full enteral feeding.

^a^ Adjusted for twin pairs and the area of hospitals as random effects.

^b^ Adjusted for maternal advanced age, GDM, HDP, ART, chorioamniotic sac type, GA, infant gender, infant's complication (BPD, NEC, PDA, neonatal sepsis), start days of FEF, duration of hospital stays, with twin pairs and the area of hospitals as random effects.

Furthermore, Supplementary Figure S3 illustrates the results of a smooth curve fitting based on a GAM model. The smooth curve fitting between BWD and EUGR revealed an inverted U-shaped relationship, with a turning point around 34.8% BWD (EDF = 3.00, *P* = 0.007). Before this turning point, the risk of EUGR increased with the increase in BWD. After reaching the turning point, further increases in BWD led to a decreased risk of EUGR. The maximum likelihood method identified the turning point of the smooth curve at BWD = 34.8%, adjusted for confounders. Additionally, the BWD value at which the curve intersected the horizontal axis was 20% for EUGR, which was considered the significant threshold for the dichotomous variable.

### SGA on the association between BWD and EUGR

The significant interaction *P*-value (<0.001) for the association of BWD across EUGR was found. In the stratified analyses (Table 3), we found a positive significant association (OR = 1.38, 95% CI: 1.30–1.47, *P* < 0.01) between the cut-off levels of BWD and EUGR among SGA infants. While we did not find similar associations among infants of non-SGA (OR = 0.75, 95% CI: 0.49–1.14).

**Table 3:** Modification of SGA on the associations between the cut-off level of BWD and individual EUGR.

SGA (yes/no)	EUGR (%)	BWD >20%		*P*_interaction_
		OR (95% CI)	*P*-value	
Stratified by SGA				
SGA infant (*n* = 632)	574 (90.82)	1.38 (1.30, 1.47)	<0.01	<0.001
NON-SGA infant (*n* = 1, 864)	563 (30.20)	0.75 (0.49, 1.14)	0.18	

Adjusted for maternal advanced age, GDM, HDP, ART, chorioamniotic sac type, GA, infant gender, infant's complication (BPD, NEC, PDA, neonatal sepsis), start days of FEF, duration of hospital stays, with twin pairs and the area of hospitals as random effects.

BWD, birth weight discordance; EUGR, extrauterine growth restriction; OR, odds ratio; SGA, small for gestational age.

### Sensitivity analyses

As Supplementary Table S1 shows, the association between the cut-off levels of BWD and EUGR remained robust when restricting to infants born with a GA of 32–36^+6^ weeks or DCDA, or infants whose mothers were of advanced age, or without GDM, or without HDP. The output of ordinal regression with random effects analysis was computed to analyze the association between BWD and EUGR group (Supplementary Table S2). Compared with the BWD<15% group, twins with the highest BWD (>25%) had a higher risk of being EUGR (AOR = 1.49, 95% CI 1.11–1.99, *P*_for trend_<0.01) after adjusting for confounders. Moreover, compared to a BWD of ≤15%, the adjusted OR of Δ*z*-score for a BWD >25% was 0.09 (95% CI: 0.02, 0.16), with a significant p value for trend (*P*_for trend_ <0.01) (Supplementary Table S3).

## Discussion

In this national multicenter study of twins, we found that each 1% increase in BWD was associated with a 1.02-fold increase in the risk of EUGR, with an adjusted OR of 1.59 for BWD >25% compared to BWD ≤15%, and the smooth curve fitting indicated an inverted U-shaped relationship with a turning point at 34.8% BWD. The association between BWD and EUGR was significant among SGA infants but not in non-SGA infants, and remained robust under various sensitivity analyses.

The 45.55% incidence of EUGR in our study is higher than in previous Chinese reports (30.2%–43.1%) (39, 40), possibly due to differences in the study population and sample size. While some BWD may represent normal physiological variation, the precise threshold for significant BWD remains controversial, with current thresholds ranging from 15% to 30% (8, 41). Our research is the first to use EUGR as an outcome indicator to explore the threshold of BWD, revealing that when BWD is greater than 15%, a higher BWD is positively associated with individual EUGR among NICU twins after adjusting for confounders. Moreover, we identified a significant threshold of 20% for BWD, consistent with previous studies (42). Different thresholds have been proposed to define high discordance as a predictor of neonatal morbidity and mortality. The American College of Obstetricians and Gynecologists considers 20% BWD as significant growth discordance (43), while the National Institute for Health and Care Excellence suggests a cutoff of 25% (44). These varying thresholds may be due to the heterogeneity of study populations and neonatal outcomes. Furthermore, our results showed a non-linear relationship between BWD and EUGR, with an inflection point at 34.8% BWD, indicating that the correlation is meaningful when BWD is below this threshold. This finding may be influenced by the smaller sample size for BWD >34.8% (*n* = 90, 3.61%), as supported by previous epidemiological studies showing that twins with higher BWD are at increased risk of fetal death (7, 45, 46).

Although the mechanism linking BWD and EUGR is not well-established, a comparable “two-hit process” of metaflammation may be identified. While unequal placental sharing plays an indispensable role in the pathophysiology of BWD (47), the associated placental malperfusion can trigger fetal growth restriction through pathways like oxidative stress and inflammation (48). We speculate that this initial insult (49, 50), combined with the postnatal challenges of prematurity and the NICU environment as a second hit, contributes to EUGR in infants who experienced BWD.

Existing evidence on growth patterns among twins with BWD and SGA remains limited and inconclusive. In this study, we identified a statistically significant interaction between SGA and BWD, specifically noting that infants with SGA exhibited more pronounced detrimental associations between BWD and EUGR. Hence, our findings align with Yang et al.'s cohort study of 150 twin pairs, which linked BWD and SGA to disordered gut microbiota and later metabolic health (51), noting that Oscillospira (enriched in SGA infants) is associated with leanness or lower weight in later life (19, 52). Additionally, SGA infants among BWD twins, who had a higher risk of neonatal morbidity during hospitalization (53), may impair nutrient intake and elevate EUGR risk (40, 54). Placental and maternal factors in BWD disproportionately restrict growth in the smaller twins, as reported by Puccio et al. (20), further explaining the differential effect of BWD on EUGR in SGA vs. non-SGA infants.

To date, no international consensus regarding the ideal growth pattern for preterm infants (55). According to previous studies, it is safe and feasible to use the traditional definition of EUGR (<10th percentile for corrected GA) as a growth aim before determining more accurate targets through large-scale studies (12, 14, 56). To prevent EUGR, expert consensus on the growth development monitoring and nutritional management of preterm infants have been issued by both the European Society of Pediatric Gastroenterology, Hepatology and Nutrition, and the Preterm Committee of Neonatology Branch of Chinese Medical Doctor Association (57, 58). However, these guidelines only classify risk levels based on GA, birthweight, or postnatal complications, without considering twins, who had higher risk of lower GA, lower birthweight, or more postnatal complications. A recent review suggested that SGA infants of multiple pregnancies should be distinguished in future guidelines and that specific nutrition recommendations for appropriate weight gain should be given (59). Our results further provided comprehensive evidence regarding the association between BWD and the risk of EUGR, as well as the modifying effect of SGA. By controlling for important confounders and conducting multiple sensitivity analyses based on national data, our findings indicate that twins with different BWD, as well as infants with or without SGA, exhibit different growth patterns. These observed differences point to potential underlying variations in metabolic needs and justify future research to determine if targeted clinical interventions and individualized care strategies would be beneficial.

### Strengths and limitations

The strengths of our study include a large national sample size and comprehensive information on maternal characteristics and neonatal complications, which ensured the robustness of our findings. However, several potential limitations merit further discussion. First, in a cross-sectional survey, it is not possible to fully establish the causal nature of BWD relative to the development of EUGR, cautioning against drawing causal inferences. However, there is a temporal relationship between BWD and EUGR, which helps avoid the phenomenon of cause-effect inversion. Second, the lack of detailed data regarding factors such as fluid intake in the first week, protein energy ratio, milk administration speed, reasons for fasting, and other variables makes it impossible to comprehensively analyze the nutritional status of this study population. Although we adjusted for time to enteral nutrition in the main models, future prospective studies that collect nutritional intake of infants are needed. Third, although applying singleton-based Fenton references to twins may underestimate postnatal growth restriction, we used the Δ*z*-score to validate the association between higher BWD and a lower change in weight *Z*-score from birth to discharge. Finally, our study did not examine the correlation of BWD with linear growth or head circumference. Future studies should incorporate these critical anthropometric measures to provide a more holistic view of postnatal growth patterns in discordant twins.

## Conclusion

In this present study, BWD was associated with higher odds of EUGR in preterm twins, and the association was pronounced among SGA infants. Future well-designed with long-term follow-up researches are needed to confirm our findings.

## Data Availability

The data analyzed in this study is subject to the following licenses/restrictions: The data are available from the corresponding author on reasonable request. Requests to access these datasets should be directed to Qiliang Cui, cuiql_gysy@163.com.
