# Supplementary material for: Association between birthweight discordance and extrauterine growth restriction among preterm twins: a national multi-center study in China
Source: Front Pediatr. 2026 Jan 23;13:1709824. doi: 10.3389/fped.2025.1709824 (PMC12876194; doi:10.3389/fped.2025.1709824)
Supplement: Supplementary file 1 [file Datasheet1.docx]

**Supplementary Information**

**Fig. S1** Geographical division in China and the sampling area in the current study. (Color figure online)

**Fig. S2** The flowchart of this study

**Fig. S3** The association between BWD and EUGR in the full adjusted multivariable GAM.

**Table S1.** Association between the cut-off level of BWD with EUGR in the subgroup analysis

**Table S2** Association between BWD and EUGR group using ordinal logistic regression with random effects.

**Table S3**. Associations between BWD and individual Δz-score


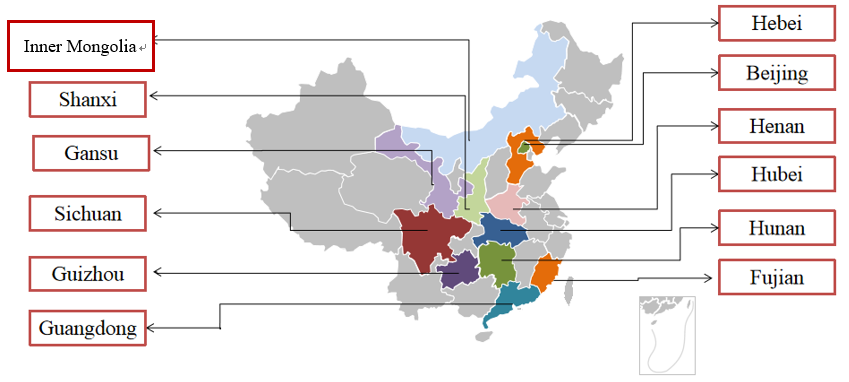


**Fig. S1** Geographical division in China and the sampling area in the current study. (Color figure online)


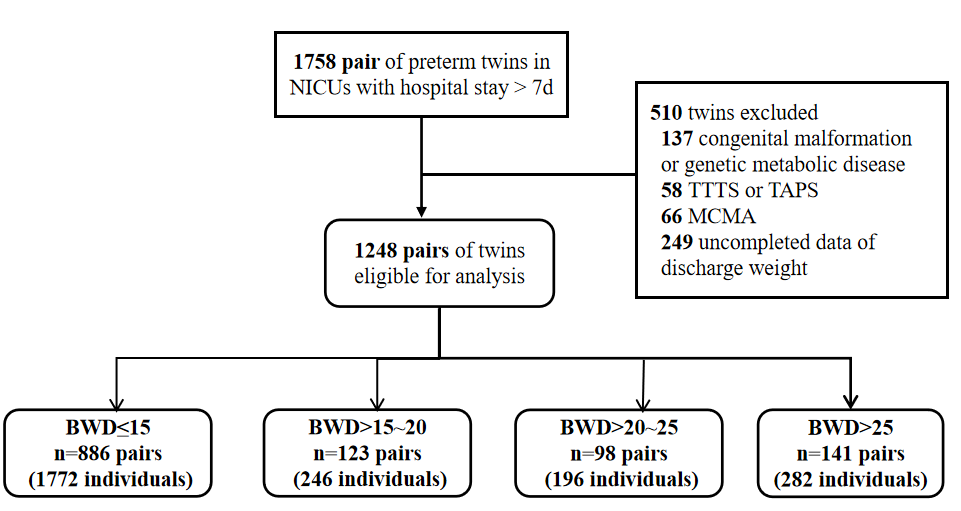


Abbreviation: NICU, neonatal intensive care unit; TTTS, twin-to-twin transfusion syndrome; TAPS, twin-anemia-polycythemia sequence; MCMA, monochorionic monoamniotic; BWD, birth weight discordance

**Fig. S2** The flowchart of this study.


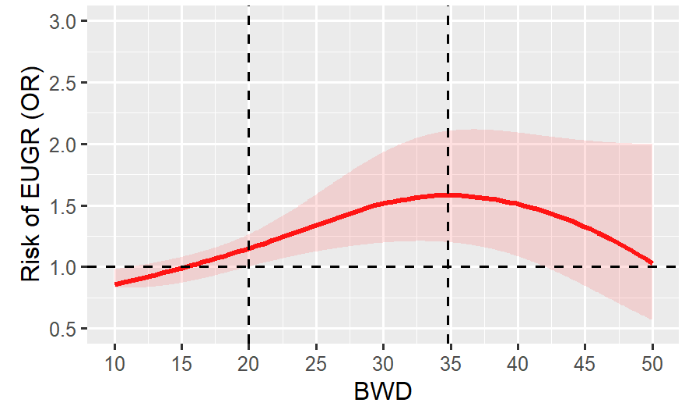


^a^

**Fig. S3** The association between BWD and EUGR in the full adjusted multivariable GAM.

The red dotted line represented the 95% CI, and the red solid line represented the estimate OR.

^a^ Adjusted for maternal advanced age, GDM, HDP, ART, chorioamniotic sac type, GA, infant gender, infant's complication (BPD, NEC, PDA, neonatal sepsis), start days of FEF, duration of hospital stays, with twin pairs and the area of hospitals as random effects.

Abbreviations: BWD, birth weight discordance; EUGR, extrauterine growth restriction; OR, odds ratio.

**Table S1. Association between the cut-off level of BWD with EUGR in the subgroup analysis**

|  | **OR (95% CI)** | ***P* value** | ***P* _interaction_** |
| --- | --- | --- | --- |
| **Overall** | 2.31 (1.50, 3.57) | <0.01 |  |
| **Maternal age** |  |  | 0.26 |
| young age | 1.39 (0.96, 2.01) | 0.08 |  |
| advanced age | 2.31 (1.20, 4.46) | 0.01 |  |
| **Chorioamniotic sac type** |  |  | 0.17 |
| MCDA | 2.12 (0.96, 4.71) | 0.06 |  |
| DCDA | 2.67 (1.56, 4.55) | <0.01 |  |
| **GDM** |  |  | 0.99 |
| yes | 1.69 (0.54, 5.30) | 0.37 |  |
| no | 1.79 (1.11, 2.88) | 0.02 |  |
| **HDP** |  |  | 0.45 |
| yes | 2.20 (0.93, 5.23) | 0.07 |  |
| no | 1.88 (1.12, 3.18) | 0.02 |  |
| **GA** |  |  | 0.51 |
| 28~31^+6^w | 0.99 (0.46, 2.10) | 0.97 |  |
| 32~36^+6^w | 1.65 (1.06, 2.57) | 0.03 |  |
| **Neonatal complications** |  |  | 0.85 |
| Yes | 1.66 (0.89, 3.09) | 0.11 |  |
| No | 1.72 (0.99, 2.99) | 0.06 |  |

Adjusted for maternal advanced age, GDM, HDP, ART, chorioamniotic sac type, GA, infant gender, infant's complication (BPD, NEC, PDA, neonatal sepsis), start days of FEF, duration of hospital stays, with twin pairs and the area of hospitals as random effects.

Abbreviations: BWD, birth weight discordance; EUGR, extrauterine growth restriction; OR, odds ratio; MCDA, monochorionic diamniotic; DCDA, dichorionic diamniotic; GDM, gestational diabetes mellitus; HDP, hypertensive disorders of pregnancy; GA, gestational age.

**Table S2. Association between BWD and EUGR group using ordinal logistic regression with random effects**

|  | **OR (95% CI)** | ***P* value** | ***P* _for trend_** |
| --- | --- | --- | --- |
| **Crude Model ^a^** |  |  | <0.001 |
| ≤15% | Ref | **-** |  |
| 15~20% | 1.37 (1.05, 1.79) | 0.02 |  |
| 20~25% | 1.55 (1.15, 2.09) | <0.01 |  |
| >25% | 2.12 (1.64, 2.75) | 0.001 |  |
| **Adjusted Model ^b^** |  |  | 0.001 |
| ≤15% | Ref | **-** |  |
| 15~20% | 1.32 (0.98, 1.77) | 0.06 |  |
| 20~25% | 1.43 (1.03, 1.98) | 0.03 |  |
| >25% | 1.49 (1.11, 1.99) | **<0.01** |  |

^a^ Ordinal logistic regression with a hospital-level random intercept was used for crude model.

^b^ Ordinal logistic regression adjusted for maternal advanced age, GDM, HDP, ART, chorioamniotic sac type, GA, twin's gender, twin's complication (BPD, NEC, PDA, neonatal sepsis), days of FEF, duration of hospital stays, with the area of hospitals as random effects.

**Table S3. Associations between BWD and individual** **Δz-score**

|  | **Crude Model^a^** | | |  | **Adjusted Model^b^** | | |
| --- | --- | --- | --- | --- | --- | --- | --- |
|  | **OR (95% CI)** | ***P* value** | ***P* _for trend_** |  | **OR (95% CI)** | ***P* value** | ***P* _for trend_** |
| **Categories of BWD** | |  | 0.39 |  |  |  | 0.01 |
| ≤15% | Ref | **-** |  |  | Ref | **-** |  |
| 15~20% | 0.03 (-0.06, 0.12) | 0.54 |  |  | 0.04 (-0.04, 0.11) | 0.31 |  |
| 20~25% | 0.04 (-0.06, 0.14) | 0.42 |  |  | 0.05 (-0.04, 0.13) | 0.27 |  |
| >25% | 0.02 (-0.06, 0.11) | 0.62 |  |  | 0.09 (0.02, 0.16) | 0.01 |  |

^a^ Adjusted for twin pairs and the area of hospitals as random effects.

^b^ Adjusted for maternal advanced age, GDM, HDP, ART, chorioamniotic sac type, GA, infant gender, infant's complication (BPD, NEC, PDA, neonatal sepsis), start days of FEF, duration of hospital stays, with twin pairs and the area of hospitals as random effects.

Abbreviations: BWD, birth weight discordance; OR, odds ratio; ART, assisted reproductive technology; GDM, gestational diabetes mellitus; HDP, hypertensive disorders of pregnancy; GA, gestational age; FEF, full enteral feeding.
